# A Systematic Study of the Structural Properties of Technical Lignins

**DOI:** 10.3390/polym17020214

**Published:** 2025-01-16

**Authors:** Keiti Gilioli Tosin, Noriê Finimundi, Matheus Poletto

**Affiliations:** 1 Postgraduate Program in Engineering of Processes and Technologies (PGEPROTEC), University of Caxias do Sul (UCS), Caxias do Sul 95070-560, Brazil; kgtosin@ucs.br; 2 Exact Sciences and Engineering, Chemical Engineering, University of Caxias do Sul (UCS), Caxias do Sul 95070-560, Brazil; nfinimundi@ucs.br

**Keywords:** lignosulfonate, characterization, hydrogen bonds, phenolic hydroxyl groups, combustion

## Abstract

Technical lignins are globally available and a sustainable feedstock. The unique properties of technical lignins suggest that these materials should have several industrial applications. The main proposal of this study is to evaluate the relationship between the structure and properties of two technical lignins. Morphological, chemical, physical, and thermal properties of sodium lignosulfonate (LGNa) and magnesium lignosulfonate (LGMg) were investigated. The results showed that a higher formation of intramolecular hydrogen bonds may occur in lignins with a higher content of phenolic hydroxyl groups, such as LGMg. As a result, an increase in the energy of hydrogen bonds in the lignosulfonate structure was observed, without significant change in the hydrogen bond distances. In addition, higher content of phenolic hydroxyl groups might also reduce lignosulfonates thermal stability. The combustion index value was three times higher for LGMg than for LGNa. The characterization study also revealed that phenolic hydroxyl groups influence the main properties of technical lignins and can be a determining factor when these lignosulfonates are used in industrial applications.

## 1. Introduction

Lignin and its derivatives are widely recognized as a potential feedstock for the future production of chemicals, materials, and renewable energy, especially because these industries try to reduce the utilization of fossil fuels [1,2,3]. About 3 × 10^11^ metric tons of lignin and its derivatives are annually available and another 2 × 10^10^ are biosynthesized per year [4]. To obtain lignin commercial value-added products, lignin is isolated from biomass by means of several methods [4,5]. Kraft and sulfite pulping processes are widely used, due to their effective lignin separation from wood [5]. Technical lignins are obtained as byproducts from these pulping processes [5,6], named kraft lignins and lignosulfonates, also known as sulfonated lignins. However, lignins face a great challenge in their technical valuation due to their highly complex and variable structure [6]. The molecular weight, polydispersity, and functional groups also vary among the different types of technical lignins [4]. However, most of them are burned for thermal recycling [5], and only minor parts are used as commercial materials, such as lignosulfonate as a dispersant [4].

Lignosulfonate accounts for almost 80% of the technical lignins in the world market [7,8]. The annual worldwide production of lignosulfonates is approximately 1.8 million tons [6]. Environmental concerns have been the driving force to change the current scenario in favor of the large utilization of technical lignins in industrial applications. Four main functions of lignosulfonate have been explored for industrial applications [7]. These functions include adhesive properties for a binder, plastic properties for a dispersant, surface-stabilizing properties for an emulsifier, and chelation properties for a sequestrant [5,7]. Lignosulfonate has also been used for other applications, such as a dispersant in concrete, a component in coal briquettes, and a dispersant for dyes and pigments, flocculants, and metal absorbents, among others [5,7]. Lignosulfonates can be used as corrosion inhibitors in a potable water distribution system [5]. The complex efficiencies of lignosulfonates at 100 gL^−1^ dosage are 95% with iron and copper and 70% with zinc [5]. Shukla et al. (2019) demonstrate that samples with 30% *w*/*v* of sodium lignin sulfonate-treated cotton fabric presented a self-extinguishing behavior, while control cotton fabric was found to burn out with a flame and afterglow [9]. Pang et al. (2018) observed that nitrogen-containing hierarchical porous carbon spheres derived from sodium lignosulfonate generates high-performance supercapacitors and is therefore a promising candidate for the field of energy storage [10].

Lignosulfonate consists of a hyper-branched and highly cross-linked macromolecular structure, which has both hydrophobic groups and hydrophilic terminal groups such as sulfonic acid groups [8]. This combination confers high solubility in water, favoring its dispersion. In addition, lignosulfonate maintains a basic three-dimensional structure similar to lignin, which can physically prevent aggregation and allow immobilization [6,8]. Furthermore, during the pulping process, Na^+^ and Mg^2+^, among other cations, can replace hydrogen in the sulfonic group, depending on the type of sulfite salt used in conjunction with sulfonic acid, generating different types of lignosulfonates [6]. Lignosulfonate is often mixed with polymeric materials, which can result in an improve of composite mechanical properties [8]. Because of its structure, it is often used as a bio-based polymer material [3,9]. Due to the abundant presence of sulfonic and hydroxyl groups in its structure, lignosulfonate demonstrates an affinity for metal ions [11]. This feature enables the creation of environmentally friendly and low-cost adsorbent composites [6].

Quantitative analysis methods are used to characterize both natural and technical lignins. Several spectroscopy methods are often used to investigate the properties of technical lignins. UV spectroscopy is extensively used to evaluate the main structural groups presented in lignin and technical lignins [12,13,14,15]. Fourier Transform Infrared Spectroscopy analysis (FTIR) is commonly used qualitatively, for example, to identify different functional groups [5]. Furthermore, the thermal properties of lignin can be investigated by thermogravimetric analysis [16,17].

Lancefield et al. (2019) realized that total reflection analysis (ATR)-FTIR of lignins, combined with multivariate analysis techniques, based on principal component analysis (PCA) and partial least squares modeling (PLS), can provide a wealth of structural information [5]. Thus, they showed that this technique can be used to predict the binding abundance between lignin techniques with high accuracy. Blindheim et al. (2023) present complementary insights into the evaluation of the structure and properties of technical samples of lignin via FTIR [18]. The FTIR technique could provide satisfactory and detailed results in the characterization and quantification of functional groups presented in the technical lignins structures [18]. Wang et al. (2018) used thermogravimetry to compare the lignin fractions [19]. The authors observed that both lignins studied presented a two-stage thermal degradation [19]. According to the authors, molecular weight and differences in chemical structure of the different lignin fractions may be responsible for variations in their thermal stability [19]. Suota et al. (2021) compared the thermal behavior of lignin from hardwood (LFSL) and softwood (LFHL) using thermogravimetry [20]. A similar degradation profile was observed for both samples and three well-defined thermal events were observed by thermal analysis [20]. The authors also observed that softwood lignins generally have higher thermal resistances than hardwood lignins regardless of the requirement procedure [20].

Valorization of technical lignins is essential to bio-based economies because lignin is the only renewable aromatic polymer produced on a bulk scale [7]. A deep knowledge of the properties of technical lignins can contribute to developing better lignin-based materials. It is important to characterize technical lignins to determine how they behave in various potential applications [21]. However, the literature lacks work that evaluates the main properties of technical lignin and its relationship with structural parameters, such as the energy of hydrogen bonds, hydrogen bond distances, and combustion parameters. So, this work evaluated the properties of two technical lignins to better understand their characteristics as potential materials for the development of high-added value composites and also their conversion into functional materials based on the unique properties of lignin derivatives.

## 2. Materials and Methods

### 2.1. Materials

The lignosulfonates characterized were sodium lignosulfonate (Ultrazine Na) and magnesium lignosulfonate (Borresperse 390) both with a mean particle size of 5 µm supplied from Borregaard Linotech, São Paulo, Brazil. The samples were oven-dried at 80 °C for 8 h before the tests.

### 2.2. Sample Characterization

The morphology of each technical lignin was investigated using SEM—FEG in a Tescan Mira 3 (Brno, Czech Republic) at 15 kV. Energy dispersive spectroscopy (EDS) analysis was also used to determine the elements presented in both technical lignins studied. Density values were obtained based on ASTM D-297. A pycnometer Gay-Lussac pattern, calibrated with capacity for 25 mL was used. Lignosulfonate samples of up to 1 mg in triplicate using ethanol was used as the solvent.

UV spectroscopy analysis was carried out in a spectrophotometer Thermo Spectronic Genesys 10 UV (Rochester, NY, USA) in the range of 200–400 nm. Ionization difference ultraviolet spectroscopy was used to estimate the content of free phenolic hydroxyl groups in both lignosulfonates studied. The method adopted in this work was previously described in the literature [12,14,22] and is based on the absorption difference between phenolic units in neutral (H_2_O) and alkaline (NaOH 0.001M) solutions.

Fourier transform infrared spectroscopy (FTIR) analysis was performed in attenuated total reflection (ATR) mode on a Nicolet IS10 Thermo Scientific spectrophotometer (Seattle, WA USA). The spectra were obtained using 32 scans, at a resolution of 4 cm^−1^, in the range of 4000 cm^−1^ to 400 cm^−1^. Second derivative spectra were obtained by applying the Savitzky–Golay function to determine the energy of the hydrogen bonds and hydrogen bond distance for technical lignins studied.

Thermogravimetric analysis (TGA) of the technical lignin samples was performed using Shimadzu equipment, model TGA—50 (Kyoto, Japan). Samples of up to 10 mg were subjected to analysis in an air atmosphere with a gas flow of 63 mL/min, a temperature range of 25 °C to 800 °C, and a heating rate of 10 °C/min.

## 3. Results and Discussion

### 3.1. Morphological and Physical Characterization

**Figure 1:**
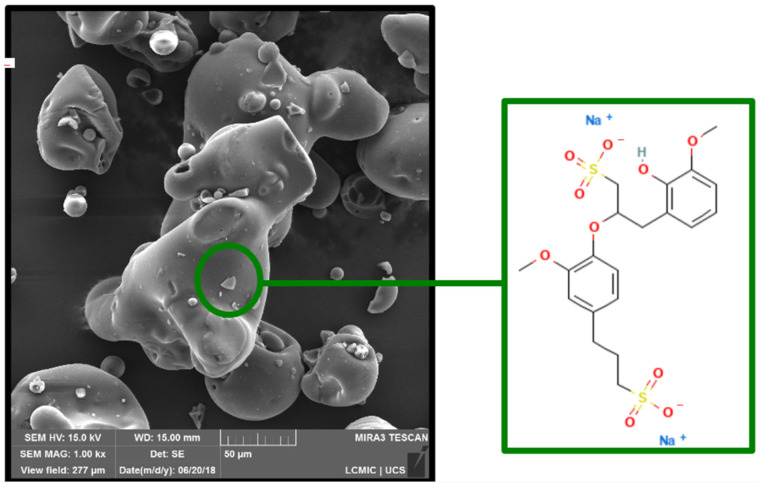
SEM of LGNa with the structure of sodium lignosulfonate (Source: PubChem, 2009).

**Figure 2:**
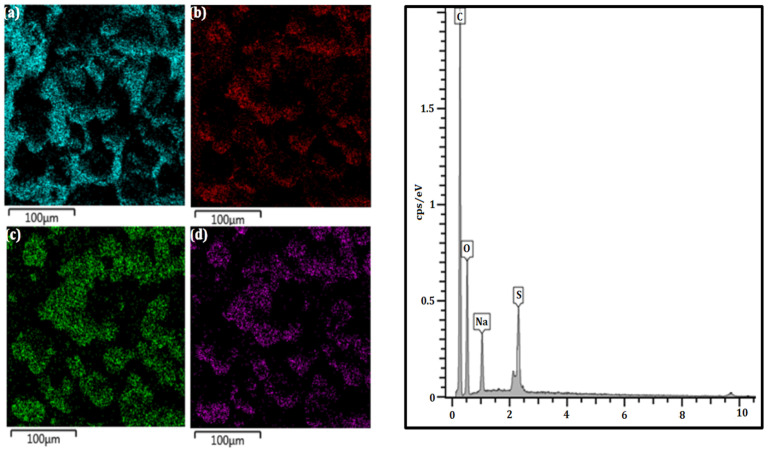
Mapping of elements (**a**) C, (**b**) O, (**c**) S, and (**d**) Na in the LGNa and EDS spectrum.

The morphology of LGNa with a magnification of 1000× can be seen in Figure 1. The mapping of elements in the LGNa and the EDS spectrum can be visualized in Figure 2.

**Figure 3:**
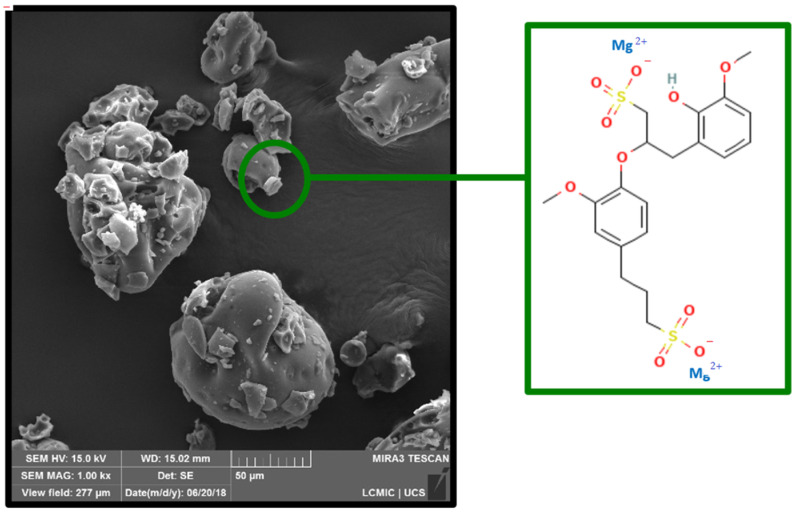
SEM of LGMg with the structure of magnesium lignosulfonate (Source: adapted from PubChem, 2009).

**Figure 4:**
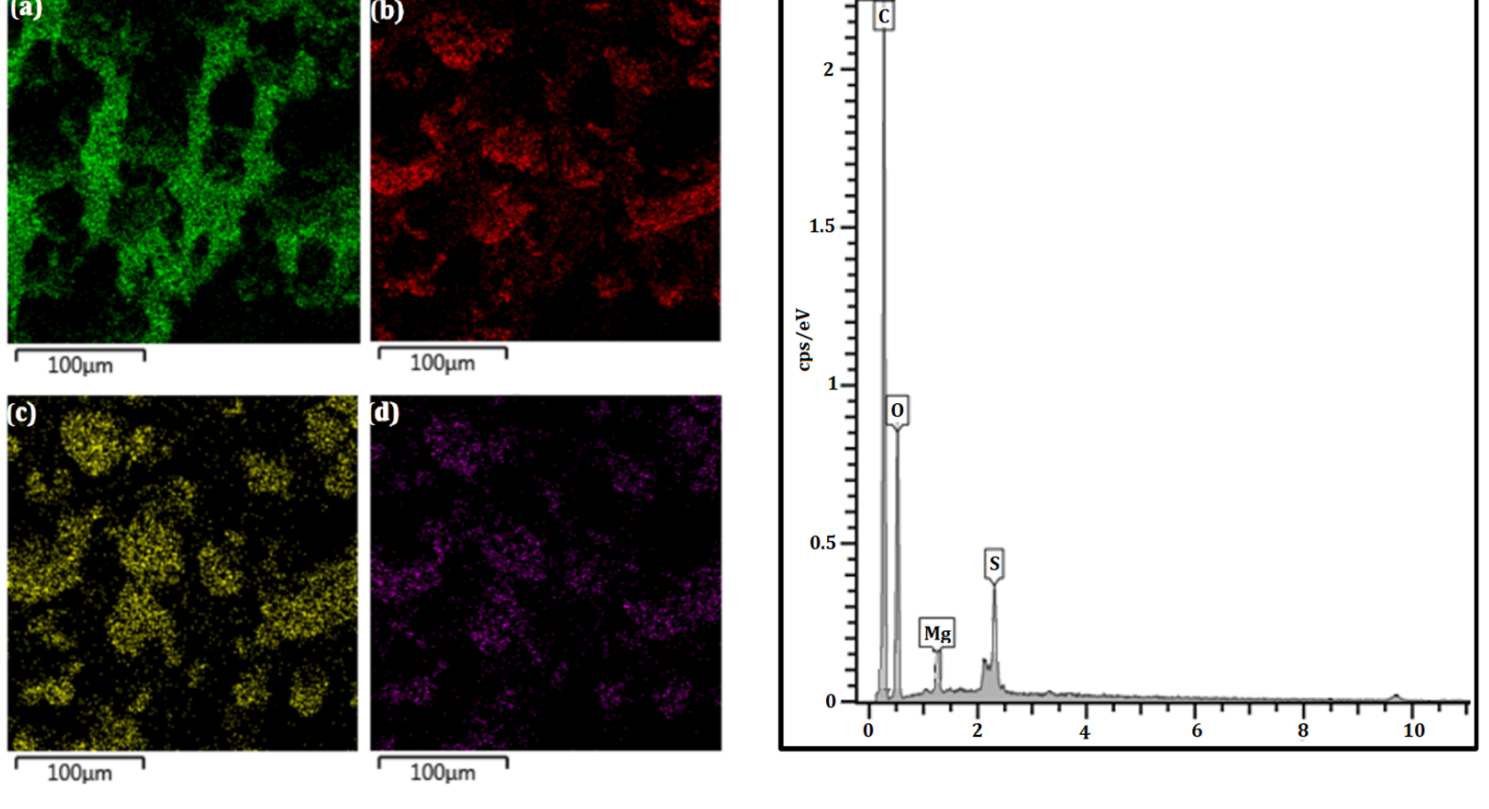
Mapping of elements (**a**) C, (**b**) O, (**c**) S, and (**d**) Mg in the LGMg and EDS spectrum.

The morphology of LGMg with a 1000× magnification is depicted in Figure 3. Visualization of element distribution in LGMg, along with the EDS spectrum, is shown in Figure 4.

**Figure 5:**
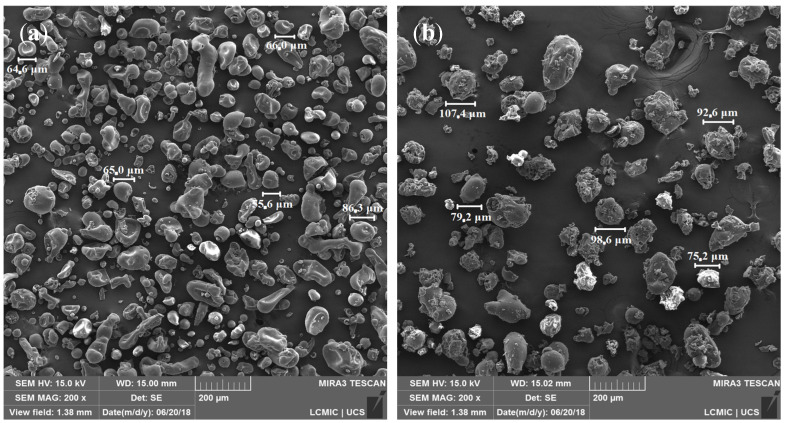
Particle sizes of LGNa (**a**) and LGMg (**b**) with magnification of 200×.

For both technical lignins, the chemical elements were distributed similarly in the EDS spectrum, with the differentiation of Na^+^ and Mg^2+^ cations. The lignosulfonates presented the highest percentage of carbon followed by oxygen and sulfur. The obtained result are in agreement with others results from the literature, such as in the study of lignins by Han et al. (2019) [23], Suota et al. (2021) [20] and Shukla et al. (2019) [9]. The particle shapes of both samples are also similar. In general, both technical lignins were characterized by spherical and hollow particles, presenting a wide particle size distribution, as can be seen in Figure 5. However, LGMg has larger particles with an average diameter of around 91 µm, while for LGNa the particle diameter remains at around 68 µm. Angelini et al. (2019) [12] and Lemes et al. (2010) [24] also verified that lignosulfonates were formed by spherical and hollow particles, but with a wide particle distribution, from 17 to 90 µm.

The density of LGNa and LGMg was calculated according to ASTM 297. Thus, the LGNa sample resulted in a density of 1.135 g/cm^3^ ± 0.003, while the value for LGMg is 1.240 g/cm^3^ ± 0.008. The differences in density between the samples may be attributed to the sizes of the Na^+^ and Mg^2+^ cations. LGMg exhibited a higher density value when compared to LGNa, possibly due to the greater atomic mass of Mg compared to Na. Additionally, the structure is another parameter to be considered, as magnesium compounds typically have a more compact structure compared to sodium compounds. In addition, the particle sizes of the samples present a difference, as can be seen in Figure 5.

Considering practical industrial applications, the differences in particle size and density can have some advantages and disadvantages. When these lignosulfonates are used in composite formulations, the lower particle size of LGNa may generate a larger superficial contact area with the polymer matrix, without excessively increasing composite density, due the lower LGNa density. However, its small particles may promote filler agglomeration in thermoplastic commodity polymers, which will probably reduce composite mechanical properties. On the other hand, the lower density of LGNa may indicate that this lignosulfonate has higher porosity than LGMg. Pang et al. (2018) observed that after pyrolysis, sodium lignosulfonate generates carbon spheres with mesopores diameter varying from 2 to 4 nm [10]. These porous carbon spheres show potential to be used as supercapacitors [10]. Other commercial lignosulfonate applications involve its usage as a dispersant. Once again, the lower density of LGNa may help to promote dispersion, without excessively increasing mixture density. Megiatto Jr. et al. (2016) studied the performance of sodium lignosulfonate as a sustainable dispersing agent for aqueous alumina colloids [25]. The authors introduce 25 mg of sodium lignosulfonate in a total alumina suspension of 100, which is equivalent to 250 ppm of lignosulfonate in the suspension [25]. As a result, the viscosity of the suspension significantly reduced by about 70% [25]. This behavior is probably associated with the rigid and negatively charged structure of the lignosulfonate molecules anchored at the alumina surface that might create both steric and electrostatic barriers that mitigate alumina particle aggregation, reducing mixture viscosity [25].

### 3.2. UV Spectroscopy

**Figure 6:**
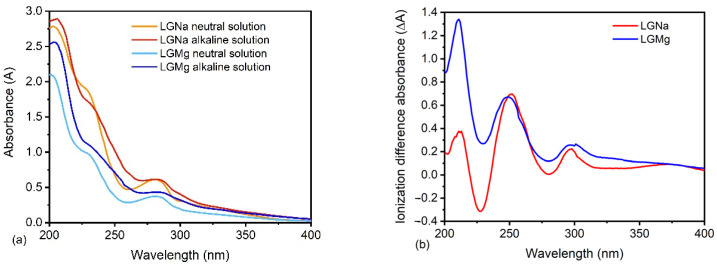
UV spectra (**a**) and ionization difference spectra (**b**) of both technical lignins evaluated.

The electromagnetic spectrum for lignin UV spectroscopy extends from 200 to 380 nm [22]. The energy absorbed in this region causes changes in the electron energy, which generates transitions of valence electrons in lignin molecules [22]. These transitions are related to the excitation of an electron from a filled molecular orbital, commonly a bonding π-orbital to the next higher energy orbital (characterized by an antibonding π-orbital), denominated as π-π* [21,22]. The UV spectrum of both technical lignins in neutral and alkaline solutions is shown in Figure 6a. The spectrum shows a typical behavior of softwood lignins [22], with a sharp peak at 210 nm, followed by a shoulder at 230 nm and a maximum peak at 280 nm. Guaiacyl compounds associated with softwood lignins present absorption bands in three regions: 200–208 nm, 227–233 nm, and 268–287 nm [12,13,14,15,22]. However, is not possible to indicate if the lignosulfonates studied derived from softwood, hardwood or non-wood species. The main absorption at 280 nm originates from the aromatic rings and non-conjugated phenolic groups presented in the structure [26] of the technical lignins.

The ionization difference spectra are normally used in lignin spectroscopy as a way of measuring phenolic content in lignin and its derivatives [12,22]. The spectra were obtained by the subtraction of the absorption curves of a compound before and after a chemical treatment. In this case, the absorption difference was between phenolic units in water and sodium hydroxide solution. The ionization difference spectra exhibit three maximum peaks located at 210, 250, and 298 nm and a wider peak at around 370 nm. The absorption band at 298 nm is probably related to hydroxyl phenyl propanoid units with saturated side chains [14,22], while the absorption at 370 nm is possibly associated with p,p′-dihydroxy-stilbenes [14,22]. The bands at 210 and 250 nm are possibly related to the absorption of the guaiacyl compound as discussed earlier.

The presence and content of phenolic hydroxyl groups in technical lignins directly affect its reactivity [21]. The activation of the aromatic ring in o-position and the ability of units with free phenolic hydroxyl groups to form intermediate units, which are susceptible to nucleophilic reaction at the benzylic carbon atom [21] are the main factors in determining the content of phenolic hydroxyl groups. In addition, the presence of a phenolic hydroxyl group tends to increase the reactivity of lignin or lignosulfonate when they are used for phenolic resin formulations as a source of phenol groups [21].

The quantitative evaluation of free phenolic hydroxyl groups in LGNa and LGMg was obtained by difference ionization UV spectroscopy in neutral and alkaline solutions. It was found that the content of phenolic groups in LGNa is 0.90 wt% (0.53 mmol/g) while LGMg presented a higher value of 1.16 wt% (0.68 mmol/g). This result implies lower reactivity for LGNa when compared with LGMg. Alonso et al. (2001) reported phenolic hydroxyl content for softwood sodium lignosulfonate equal to 0.84 wt%, while the content for lignosulfonates derived from hardwood and softwood species varies between 0.99 and 1.54 wt% [27]. Angelini et al. (2019) suggest that lower phenolic hydroxyl values, generally lower than 1 wt%, result in lower reactivity and lignosulfonate may behave as an inert or deteriorating agent towards urea-formaldehyde resin [12]. The higher number of active sites in lignin and technical lignins contributes to the preparation of lignin-based phenol-formaldehyde products [28] under alkaline conditions, such as adhesives, chelating resins, and ion exchange resins [21].

LGMg presented higher ionization difference absorbance than LGNa, as can be seen in Figure 6b. It is possible that LGMg presented highly cross-linked condensed units that generated ionization resistance resulting in higher ionization difference absorbance. However, phenolic OH from uncondensed (terminal) units would be more easily accessible to react with others lignosulfonate molecules or other compounds that result in higher lignosulfonate reactivity. Mansouri and Salvadó (2006) pointed out that the number of phenolic hydroxyl groups essentially influences reactivity of technical lignins [21]. The presence of free (terminal) phenolic hydroxyl groups tends to increase the reactivity of technical lignins towards formaldehyde when technical lignins are used in phenolic resin formulation. So, LGMg presented higher potential than LGNa to be used in phenolic resin formulations, based on its higher content of phenolic hydroxyl groups. In addition, a high phenolic hydroxyl content is also desirable when lignin and its derivatives are used in the polymerization reaction in wood adhesives [21].

### 3.3. Fourier Transform Infrared Spectroscopy (FTIR)

**Figure 7:**
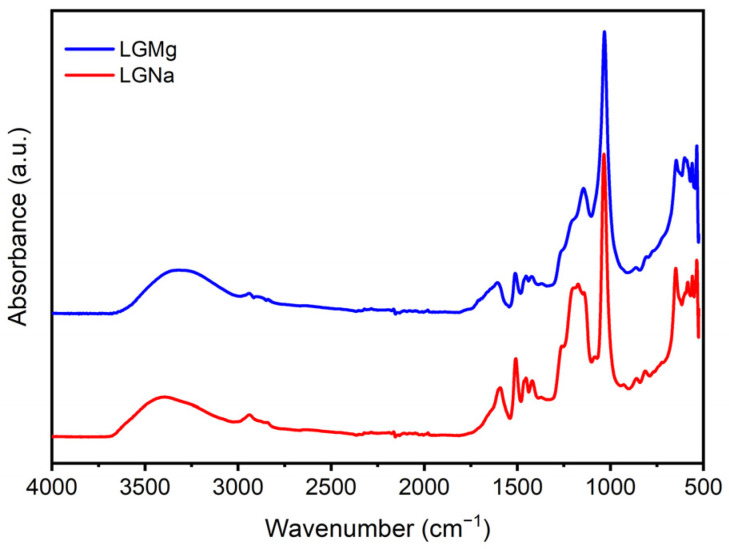
FTIR spectra of LGNa and LGMg.

The FTIR spectra of both technical lignin samples can be seen in Figure 7. FTIR spectra reveal a similar structure between each type of lignosulfonate. The FTIR spectra differ only by the band intensities, where some bands are very intense or very weak in the same region.

The band centered at around 3450 cm^−1^ is mainly associated with the hydroxyl groups [29] present in the technical lignin structure. Yan et al. (2019) state that lignosulfonate has abundant hydroxyl groups (OH) in its structure, a fact that can be proven by analyzing the spectrum of the samples in the most intense band between 3700 and 3000 cm^−1^ [30]. According to Han et al. (2019), a broad band between 3700 and 3000 cm^−1^ also indicates the presence of hydrogen bonding that may influence the thermal properties of lignins [23]. The two weak bands at around 2900–2950 cm^−1^ and 2850–2900 cm^−1^ were assigned to CH stretching in aromatic methoxyl groups and also in aliphatic methyl and methylene groups of side chains [31] of technical lignins evaluated. In the fingerprint region (1800–500 cm^−1^), no bands were observed for both technical lignins between 1715 and 1705 cm^−1^, which are associated with unconjugated carbonyl-carboxyl stretching [26].

The band between 1610 and 1595 cm^−1^ was associated with C=C stretching of the aromatic ring in the syringyl unit, while the next band at around 1515–1507 cm^−1^ occurs due to the C=C stretching of the aromatic ring in the guaiacyl unit [9,32]. There are observed differences in the intensity of these bands for both samples. LGMg presented a large, but not intense band at 1610–1595 cm^−1^ and a medium band at 1515–1507 cm^−1^. However, for the LGNa sample, both intense bands were observed at 1597 cm^−1^ and 1507 cm^−1^. This result indicates differences in the molecular architecture of both technical lignins studied due to different linkages between the phenolic hydroxyl, syringyl, and guaiacyl units that make up the lignosulfonates. LGNa presented a large band between 1250 and 1100 cm^−1^, centered at 1170 cm^−1^, probably associated with C-H in the plane deformation of guaiacyl ring units and aromatic C-H in the plane deformation of guaiacyl units [26,32]. A band at 1143 cm^−1^ is observed for LGMg and can be attributed to aromatic C-H in-plane deformation of guaiacyl units [33]. Both lignosulfonates presented an intense band at 1032 cm^−1^ associated with aromatic C-H in-plane deformation and C-O deformation in primary alcohols [23].

The hydrogen bond is essential to stabilizing the three-dimensional structure of lignin and other biological macromolecules [34]. The energy of hydrogen bonds (*E_H_*) was evaluated according to Equation (1) [35]:

(1) $$E_{H}=\frac{1}{k}\;\left[\frac{\left(v_{0}-v\right)}{v_{0}}\right]$$

where *v*_0_ refers to unbonded –OH groups (3650 cm^−1^) [36] and *ν* represents the frequency of bonded –OH groups. The constant *k* maintains the value of 2.625 × 10^2^ kJ.

To determine the hydrogen bond distances *R*, Equation (2) developed by Pimentel and Sederholm (1956) [37] was applied as follows:

Δ*v* (cm^−1^) = 4430 × (2.84 − R) (2)

In this equation, *Δv* represents the difference between *v*_0_ and *ν*, where *v_0_* is the monomeric OH stretching frequency, considered as 3600 cm^−1^, and ν is the observed stretching frequency in the infrared spectrum of the sample [36,38].

The energy of hydrogen bonds and hydrogen bond distances for the wavenumbers considered in this study and obtained from second derivative spectra are presented in Table 1. The absorption band at around 3570 cm^−1^ is associated with intramolecular hydrogen bonds in a phenolic group in lignin [36], while the absorption band at 3423 cm^−1^ is attributed to hydroxyl groups presented in aliphatic and phenolic structures [32].

The *E_H_* values for LGMg were higher than the values obtained for LGNa, for both wavenumbers evaluated. This result is in agreement with the higher content of phenolic groups in LGMg than LGNa observed in UV spectroscopy since the energy of hydrogen bonds increases with the phenolic-OH content [32]. A higher number of phenolic hydroxyl groups may promote the formation of a higher amount of intramolecular hydrogen bonds that generate the increase in *E_H_* values. Popescu et al. (2007) [39] evaluated the *E_H_* values in Eucalyptus wood at 3567 cm^−1^ and 3432 cm^−1^ and obtained 5.86 kJ and 16.23 kJ, respectively.

The *R* values, presented in Table 1, are the same for the wavenumbers evaluated for both technical lignins. Almost the same values were obtained for the same evaluated wavenumbers for hardwood and softwood in previous studies [36,39]. Zhu et al. (2022) studied the formation of hydrogen bonds in lignin using computational models. The authors tested six computational geometrical characteristics for hydrogen bonds of lignin β-O-4 models [34]. The computational models result in hydrogen bond distances varying from 2.646 Å to 2.927 Å [34], which are in agreement with the values found in this work using FTIR analysis.

### 3.4. Thermogravimetric Analysis (TGA)

**Figure 8:**
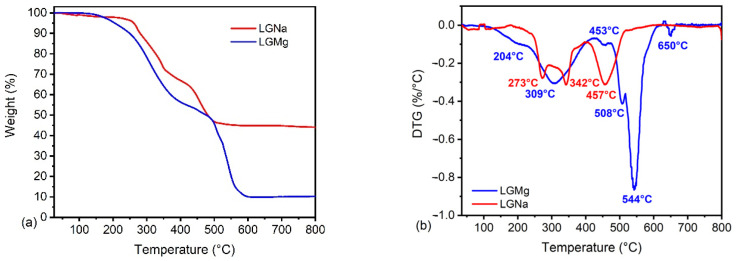
Thermogravimetric curves (**a**) and DTG curves (**b**) of LGNa and LGMg.

The structural complexity and heterogeneity of technical lignins generate several competitive and consecutive reactions during their thermal decomposition, which results in bond cleavages at different temperatures depending on the bond energies [24]. During the thermal decomposition of technical lignins, high amounts of volatile products [40] with low molar masses are released, resulting in a thermogravimetric curve with various processes of weight loss. The thermogravimetric curves and first derivative (DTG) curves of LGNa and LGMg are shown in Figure 8.

The thermal decomposition of lignosulfonate involves the production of a series of volatile compounds, that may contribute to the noise in the first and final part of the DTG curves, for both technical lignins studied. These volatile compounds may include water, carbon monoxide, carbon dioxide, and organic volatiles, such as formaldehyde, methane, and methanol [24,41]. Mercaptans and other small molecules contain sulfur, sodium, magnesium, and calcium, among others [24].

The first weight loss step at 50–150 °C can be attributed to the loss of absorbed moisture, as can be seen in Figure 8b. The second weight loss step occurs between 150 and 500 °C for both samples. At this stage, the main thermal degradation process occurred for LGNa. The major decomposition of O-containing groups and the rearrangement of the carbonaceous material may occur in this stage [25], with peaks centered at 273 °C and 342 °C, respectively. This thermal degradation is associated with the initial degradation of phenol, guaiacol, and syringol releasing alkyls, such as methane, carbon monoxide, and carbon dioxide. In addition, some sulfur and sodium-containing small molecules are also released, forming other carbonaceous structures. Another broad peak centered at 457 °C is observed for LGNa samples in Figure 8b. This fourth degradation peak may be attributed to the reaction involving sodium-containing inorganic salts and carbon, and the final decomposition of the residual oxygen-containing groups [10,25]. As can be seen in Figure 8a, the higher residual mass content for LGNa at 800 °C, when compared with LGMg, might be associated with the formation of sodium salts during the thermal degradation of its sodium-based lignosulfonate.

The second weight loss step for LGMg is initiated with a shoulder at 204 °C, followed by a broad peak centered at 309 °C ending with a small peak at 453 °C. The thermal decomposition reactions in LGMg occurred at lower temperatures than in LGNa. This behavior might be associated with the higher content of hydroxyl phenolic groups in LGMg. Higher content of hydroxyl phenolic groups at the edges of the lignosulfonate structure may make the sample more thermal reactive, initiating a thermal degradation process at lower temperatures when compared with LGNa. Thus, it is possible that decarboxylation and dehydration reactions are accelerated during the thermal degradation process. The main thermal degradation of O-containing groups and the rearrangement of the carbonaceous material occurred between 250 and 450 °C. The third weight loss step occurred between 500 and 650 °C. A small peak at 508 °C and a sharp peak at 544 °C are possibly associated with the thermal decomposition of dense aromatic ring structures [2]. The other small peak at 650 °C may also be attributed to the final thermal degradation of dense aromatic ring structures. The residual mass content at 800 °C for LGMg is lower than LGNa. The formation of magnesium salts during thermal degradation might occur in a minor amount, due to the lower magnesium content in the lignosulfonate, when compared with sodium content, as can be seen in Figure 4 and Figure 3, respectively.

TGA results were also used to evaluate the combustion parameters related to the technical lignins. The ignition temperature (*T_i_*), the burnout temperature (*T_B_*), the combustion index (*S*), the ignition index (*D_i_*), the time corresponding to the maximum degradation rate (*t_m_*), the time of ignition (*t_ig_*), the maximum rate of degradation and the mean rate of degradation were evaluated. The ignition temperature was defined, in this work, as the temperature at which the thermal degradation rate increases by 1% per minute, which is an indicator as to the starting point of the combustion process. The burnout temperature was the temperature at which the combustion rate decreased by 1% per minute, which characterized the end of the combustion process [42].

The combustion index (*S*) was calculated using Equation (3), as referenced by Protásio et al. (2019) [42]. The ignition index (*D_i_*) was determined using Equation (3), as proposed by Xiang-Guo et al. (2006) [43]:

(3) $$S=\frac{(\frac{dm}{dt})(\frac{dm}{dt}\;)me}{T_{i}^{2}\;\times \;T_{B}}$$

(4) $$D_{i}=\frac{\left(\frac{dm}{dt}\right)max}{t_{m}\;\times \;t_{ig}}$$

where “(*dm*/*dt*) *max*” is the maximum combustion rate “(% min^−1^)”, “(*dm*/*dt*) *me*” represents the mean combustion rate, “*Ti*” is the ignition temperature (°C), “*T_B_*” is the burnout temperature (°C), “*t_m_*” the time corresponding to the maximum combustion rate (min), and “*t_ig_*” the time of ignition (min).

The combustion parameters evaluated are presented in Table 2. The ignition temperature and ignition time for LGMg are lower than LGNa. The LGMg sample initiates a thermal degradation process at around 180 °C, which may represent an impediment to its use as a filler in thermoplastic polymers. The temperatures used during the processing of thermoplastic polymers normally occur at 200 °C. LGMg presented higher burnout temperature and longer time to the maximum combustion rate than LGNa. The higher volatility of LGMg may extend the time of combustion.

The maximum combustion rate and mean combustion rate are also higher for LGMg than LGNa. The higher quantity and the fast emission of volatile matter are factors that contribute to the acceleration of fuel ignition [42] and may be responsible for the higher combustion rate in LGMg. In addition, the higher content of hydroxyl phenolic groups in the LGMg structure may also contribute to accelerating the thermal degradation process. The combustion index represents the sample reactivity [42]. Higher combustion index values are associated with higher combustion intensity [42]. In general, the combustion index values are three times higher for LGMg than for LGNa. This result indicated that during thermal degradation, the LGMg sample burns with higher combustion intensity and flammability at lower temperatures and less time than LGNa, which can observed by its higher ignition index. However, in practical applications, the combustion parameters obtained in this work may change under different combustion environment, such as different oxygen concentrations, pressure, and sample conditions, among others.

## 4. Conclusions

The methods used in this work to characterize the technical lignins reveal differences in the structure and properties of both lignosulfonates evaluated. Morphological evaluation shows that both samples are formed by spherical particles with a wide particle size distribution. In the UV spectroscopy analysis, a higher content of phenolic hydroxyl groups was identified for LGMg. FTIR analysis reveals a similar structure for both technical lignins. However, the energy of hydrogen bond values for LGMg were higher than the values obtained for LGNa. A higher content of phenolic groups in LGMg than in LGNa may result in a higher amount of intramolecular hydrogen bonds that generate an increase in the energy of hydrogen bonds. Therefore, the hydrogen bond distances were not significantly affected. The structural complexity of both technical lignins studied generates three weight-loss steps during thermal degradation. LGMg presented lower thermal stability than LGNa, possibly due to the higher reactivity of the phenolic hydroxyl groups during thermal decomposition. The combustion index was three times higher for LGMg than LGNa. The presence of higher quantities of phenolic hydroxyl groups in LGMg than LGNa generates an increase in the energy of the hydrogen bonds formed, but the combustion parameters revealed a lower ignition temperature and ignition time for LGMg, which may make its usage as a reinforced polymeric composite difficult.

Technical lignins are far from being utilized to their full potential. It is necessary to understand these complex materials on both a physical and chemical level. Most importantly, applying simple characterization techniques in a sustainable lignin-derivative as a precursor to novel materials and applications is not only cost-effective and environmentally friendly but also suitable for contributing to large-scale production and utilization of lignin-based materials.

## Figures and Tables

**Table 1:** Energy of the hydrogen bonds and hydrogen bond distance for technical lignins studied.

Sample	3570 cm^−1^		3423 cm^−1^	
	E_H_ (kJ)	R (Å)	E_H_ (kJ)	R (Å)
LGNa	5.706	2.833	16.182	2.800
LGMg	5.806	2.833	16.289	2.800

**Table 2:** Values of ignition temperature (*T_i_*), burnout temperature (*T_B_*), time of ignition (*t_ig_*), time corresponding to the maximum combustion rate (*t_m_*), the maximum rate of combustion, and the mean rate of combustion (*dm*/*dt*), the combustion index (*S*), and the ignition index (*D_i_*) for technical lignins evaluated.

Sample	T_i_ (°C)	T_B_ (°C)	t_ig_ (min)	t_m_ (min)	(dm/dt)_max_ (%/min)	(dm/dt)_mean_ (%/min)	S × 10^−7^ (%^2^min^−2^°C^−3^)	D_i_ × 10^−3^ (%min^−3^)
LGNa	225.6	505.8	20.5	32.2	4.5	0.7	1.3	6.8
LGMg	179.7	586.5	16.2	52.8	7.8	1.2	4.7	9.1

## Data Availability

The original contributions presented in this study are included in the article. Further inquiries can be directed to the corresponding author.
